# Modeling the Carbothermal Chlorination Mechanism of Titanium Dioxide in Molten Salt Using a Deep Neural Network Potential

**DOI:** 10.3390/ma18030659

**Published:** 2025-02-02

**Authors:** Enhao Zhang, Xiumin Chen, Jie Zhou, Huapeng Wu, Yunmin Chen, Haiguang Huang, Jianjun Li, Qian Yang

**Affiliations:** 1 National Engineering Research Center of Vacuum Metallurgy, Kunming University of Science and Technology, Kunming 650093, China; sheeranfox@outlook.com (E.Z.);; 2 State Key Laboratory of Complex Nonferrous Metal Resource Clean Utilization, Kunming University of Science and Technology, Kunming 650093, China; 3 Beijing DP Technology Co., Ltd., Beijing 100000, China; chenyunmin@dp.tech; 4 Yunnan National Titanium Metal Co., Ltd., Chuxiong 651200, China

**Keywords:** TiO_2_, chlorination, DeePMD, AIMD

## Abstract

The molten salt chlorination method is one of the two main methods for producing titanium tetrachloride, an important intermediate product in the titanium industry. To effectively improve chlorination efficiency and reduce unnecessary waste salt generation, it is necessary to understand the mechanism of the molten salt chlorination reaction, and consequently this paper conducted studies on the carbon chlorination reaction mechanism in molten salts by combining ab initio molecular dynamics (AIMD) and deep potential molecular dynamics (DeePMD) methods. The use of DeePMD allowed for simulations on a larger spatial and longer time scale, overcoming the limitations of AIMD in fully observing complex reaction processes. The results comprehensively revealed the mechanism of titanium dioxide transforming into titanium tetrachloride. In addition, the presence form and conversion pathways of chlorine in the system were elucidated, and it was observed that chloride ions derived from NaCl can chlorinate titanium dioxide to yield titanium tetrachloride, which was validated through experimental studies. Self-diffusion coefficients of chloride ions in pure NaCl which were acquired by DeePMD showed good agreement with the experimental data.

## 1. Introduction

Due to titanium’s high specific strength, excellent corrosion resistance, and biocompatibility, it has been widely used as a high-performance lightweight metal in aerospace, defense, biomedical, petrochemical, and other high-value fields [1]. However, although titanium ranks fourth in the common metal content on Earth, the high energy consumption and cost of titanium production have limited its use in industrial and civilian applications, despite the fact that when titanium is used for mechanical systems such as civilian transport vehicles it can significantly reduce energy consumption. Therefore, exploring methods for producing low-cost titanium with excellent product quality will have a profound impact on the sustainable development of the global economy and future society [2]. Over 90% of global titanium reserves are in the form of ilmenite (TiO_2_ content ranging from 45 to 65%) [3,4]. Titanium tetrachloride (TiCl_4_) is a raw material for producing sponge titanium, titanium dioxide (TiO_2_), and nano-TiO_2_. It is also the fundamental intermediate material for the titanium-related industry [5,6]. Currently, there are two main methods for preparing TiCl_4_ [7], as follows: boiling chlorination and molten salt chlorination. Due to China’s high reserves of low-grade titanium ore [8], the process of producing titanium tetrachloride through molten salt chlorination is still under development. Molten salt chlorination is currently the most effective and mature refining process for high calcium and magnesium content-rich titanium materials [9]. The molten salt chlorination process involves complex exothermic heterogeneous (gas–liquid–solid) reactions [10].

The problem with the method of molten salt chlorination is that it is not environmentally friendly compared with boiling chlorination, as a large amount of waste salt that is difficult to recycle will be produced during the production process [11]. Therefore, it is necessary to further understand the mechanism of the carbon–chloride reaction in order to improve the production efficiency of products when generating the same amount of waste salt, which is also a way to reduce environmental pollution. This production process generates a large amount of waste salt that is difficult to recycle.

However, due to the complexity of the reaction, as well as the toxicity and corrosiveness of chlorine gas, there has been limited research on its reaction mechanism. Nevertheless, previous studies have been conducted on this topic. For example, Julio et al. [11] conducted a study in which carbon powder and titanium dioxide powder were mixed, and the chlorination process of titanium dioxide was found to occur in two reaction stages using thermogravimetric analysis. Zhu et al. [11] proposed a novel process for the treatment of high-temperature phase-changing molten salt chlorides. By introducing Na_2_SiO_3_ into the molten salt chloride, NaCl remains in a liquid state, resulting in the formation of a calcium-containing solid phase that allows for the repeated reuse of NaCl. Zhang et al. [12] carried out experiments on boiling chlorination reduction of natural rutile at temperatures ranging from 1173 K to 1273 K in a fluidized bed reactor, and investigated the kinetics and thermodynamics of this reaction. He et al. [13] conducted a study on the degradation kinetics and mechanisms of trace pollutants in the UV/chlorine/activated carbon-TiO_2_ process. Jing et al. [14] proposed a new process for the acid leaching method to manufacture raw materials suitable for boiling chlorination. Sampath et al. [15] developed several methods, such as aluminum thermal reduction and molten salt electrolysis, which can be used to synthesize TiO_2_ and other related titanium compounds from natural ilmenite ores. Hamanaka et al. [16] studied a method for recycling and reusing titanium waste and iron chloride waste using molten salt, and conducted thermodynamic research on this system. Artsdalen et al. [17] experimentally determined the density and electrical conductivity of pure sodium (NaCl) in its molten state. Janz et al. [18] measured the self-diffusion coefficient of chloride ions in molten NaCl salt. Yang et al. [19] used AIMD methods to investigate the adsorption of chlorine (Cl_2_) and carbon dioxide (CO_2_) on TiO_2_ surfaces in the fluidized chlorination process; they found that both C and CO could promote the adsorption reactions of Cl_2_ on the TiO_2_ (100) surface, and that C was more favorable to the adsorption process. In our initial work, Deng et al. [20] elucidated the influence mechanism of CaCl_2_ content on the microstructure and diffusion characteristics of molten salt, and qualitatively and quantitatively analyzed that the addition of Ca^+^ reduced the free Cl^−^ and restricted the diffusion of Cl^−^ ions. To our knowledge, there have been few reports on mechanistic studies of molten salt carbothermal chlorination at the microscale level at present. Overall, the literature that is currently available on the mechanistic studies of molten salt carbothermal chlorination method at a microscale level is limited. It is important to note that this paper simplifies the reaction model by only retaining the main species involved in chlorination reactions, namely Cl_2_, TiO_2_, C, and NaCl.

Based on density functional theory (DFT), AIMD simulations often face the dilemma of balancing computational accuracy, efficiency, and cost [21,22,23,24]. In recent years, with the continuous expansion of applications for artificial intelligence algorithms, the DeePMD simulation method has successfully combined ab initio molecular dynamics with deep learning arithmetic to address this dilemma. This DeePMD method has achieved significant results, with its accuracy and high efficiency being validated [25,26,27,28]. In a study using the DeePMD method to investigate mechanisms, Zhang et al. [29] propose that combustion encompasses thousands of reactions and hundreds of species. A neural network-based molecular dynamics (MD) simulation was employed to investigate methane combustion. The simulation unveiled intricate reaction processes and identified specific molecular species. In total, 798 reactions were documented, including novel pathways. This research signifies a new era for neural network-based MD simulations in the study of complex reaction systems. Zheng et al. [30] posited that reactive chlorine species (RCS) play a crucial role in global oxidation and disinfection processes. In their study, they developed a machine learning-based quantitative structure–activity relationship (QSAR) model utilizing quantum chemical descriptors and Morgan fingerprints as features to predict the reaction rates of RCS with organic compounds. The performance of the machine learning model surpassed that of traditional multiple linear regression models. The integration of quantum chemical descriptors and Morgan fingerprints enhanced the predictive accuracy for all four types of RCS. SHAP analysis indicated that the highest occupied molecular orbital energy, charge, and specific functional groups are key descriptors influencing these predictions. Wu et al. [31] investigated the effect of different oxygen vacancy defects in titanium dioxide on surface activity and electronic energy changes at different temperatures. Therefore, this paper first uses AIMD to conduct molecular dynamics simulations for investigating chlorination reactions before training a required DeePMD function. In addition, based on the dataset of AIMD, DeePMD simulation methods [32], which achieve simulations of atomic interactions over larger spatial and temporal scales with accuracy similar to DFT, will also be introduced to analyze microscopic structural changes of TiO_2_ during chlorination processes in molten salt.

## 2. Method

### 2.1. AIMD Method and Parameters

In this study, the structure of the molten salt chlorination reaction was constructed using the visualizer modulus in Materials Studio [33]. In order to avoid interactions between periodic structures, periodic boundary conditions were used to maintain the number of atoms and eliminate boundary effects. The model contains TiO_2_, Cl_2_, C, and NaCl (a total of 527 atoms, including 127 Na atoms, 172 Cl atoms, 92 C atoms, 58 Ti atoms, and 78 O atoms, the composition of substances in the system is based on the raw material ratios in the chlorination process of the factory), with supercell parameters a = b = 22.480 Å, c = 24.050 Å, and α = β = γ = 90°.

Subsequently, the constructed model was subjected to geometric optimization and molecular dynamic simulation using VASP 6.3 [24] (Vienna Ab-initio Simulation Package). The cutoff energy was set at 400 eV, with a maximum of 1500 steps for atomic relaxation and a convergence criterion of 1 × 10^−4^ eV for self-consistent field (SCF) calculations. Furthermore, a Nosé–Hoover thermostat [34] was employed during the simulation process using the NVT ensemble, with a convergence criterion of 1 × 10^−5^ eV for SCF and 1 × 10^−4^ for atomic relaxation, along with a maximum of 5000 steps and a broadening factor of 0.1. The system temperature was set at 1073 K, 1173 K, 1273 K, 1373 K, and 1473 K, respectively. Under NVT ensemble conditions, AIMD simulations were conducted over a duration of 5 ps with a time step of 1 fs. Periodic boundary conditions were utilized to maintain constant atom number and eliminate boundary effects throughout the simulations.

Finally, the electronic properties of the system during molecular dynamics simulations were calculated using the CASTEP module [35] in the Material Studio 2017 software package. The generalized gradient approximation (GGA) with the Perdew–Burke–Ernzerhof exchange–correlation function [36] was employed to handle the exchange–correlation interactions. The Brillouin zone was sampled using the Monkhorst–Pack scheme [37] with a 1 × 1 × 1 k-point grid. To achieve good accuracy at reasonable computational cost, the energy cutoff was set to 517 eV. The maximum displacement tolerance, energy tolerance, and maximum force tolerance were set to 0.002 Å, 0.05 eV/Å, and 2.0 × 10^−5^ eV/atom, respectively.

### 2.2. DeePMD Method Training and Validation

DeePMD [27,32,38] is a neural network-based MD simulation approach. It involves training a potential energy function using data obtained from ab initio molecular dynamics simulations, and then applying the trained function in LAMMPS [39] for further molecular dynamics simulations. In this study, we collected data from 5 sets of AIMD simulations to construct the training dataset. The DeePMD-kit 2.2.6 [32,38] software was used for data training to obtain high-dimensional potential energy functions, with the following training parameters.

**Figure 1:**
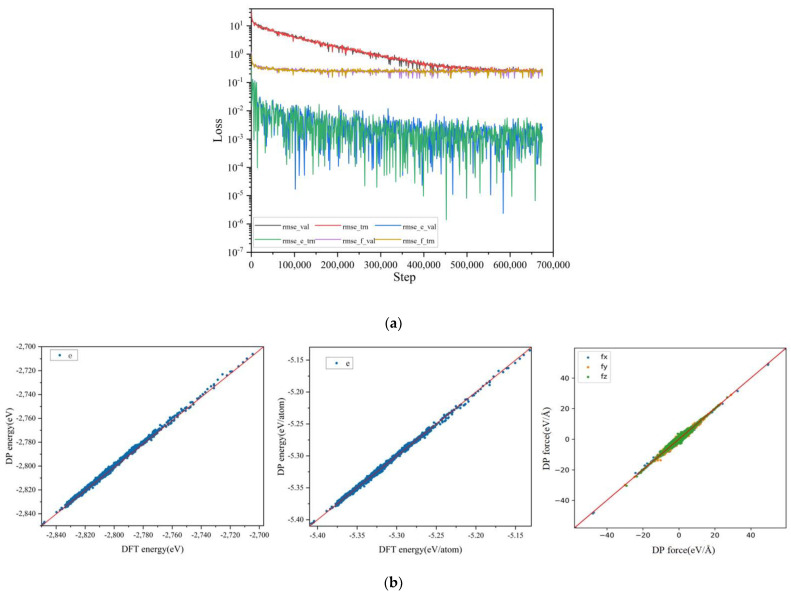
(**a**) Fitted curves of energy and force, (**b**) scatter plot of the predicted values (DeePMD) and the actual values (DFT) of the validation set.

During the training process, 90% of the sample data will be randomly sampled as the training set, and 10% of the sample data will be used as the validation set. The smooth version of Deep MD-kit2.2.3 will be employed, with the descriptor keyword “type” set to “se_a”. Based on the system characteristics, rcut_smth for expressing system interactions is set to its default value, while r_cut for atomic interaction force limit distance is set to 8 Å. The embedding neural network used for generating descriptors is set to (20, 40, 80), and the deep neural network is set to (200, 200, 200). The structure of the network model and the learning weight parameters are derived from recommended parameter values obtained through experiments conducted by DP across various systems. The learning rate parameters during training are initialized at a starting learning rate of 0.001 and an ending learning rate of 3.51 × 10^−8^; their decay type is chosen as exponential. Regarding the setting of loss functions, only energy and force loss function coefficients need to be considered due to the calculated systems. Accordingly, corresponding weight coefficients $p_{e}^{\mathrm{start}}$, $p_{f}^{\mathrm{start}}$,$p_{e}^{\mathrm{limit}}$, and $p_{f}^{\mathrm{limit}}$ at the respective start and end times are assigned values of 0.02, 1000, 1, and 1, respectively. In general, there is typically a relationship of approximately *200 between the learning rate adjustment and the training step size, so the number of training steps is specified as 700,000. Finally, the training performance can be visually inspected through the loss curve as shown in Figure 1a.

It is evident from this figure that the results of the testing set during the training process are consistent with those of the validation set. Therefore, it can be concluded that there is no underfitting or overfitting issue. Furthermore, based on the deviation magnitude of energy and force fitting from the *y*-axis, the statistical analysis of the validation results of the validation set indicates that the root mean square error (RMSE) of the force and the energy RMSE converge to a force of approximately ~10^−2^ eV/Å and an energy of ~10^−3^ eV per atom. This meets accuracy requirements and thus validates using this model for subsequent simulation work.

After the training, the model’s fitting performance can be observed through the scatter plot of the predicted values (DeePMD) and the actual values (DFT) of the validation set. It is evident in Figure 1b that the model fits the dataset very well. In the validation results of the validation set, the MAE of energy for each atom was calculated to be 1.525 × 10^−3^ eV, and the MAE of force in the three coordinate directions was approximately ~10^−1^ eV/Å.

### 2.3. DeePMD Model Construction and Parameters

Using the construction tool, a system containing TiO_2_, Cl_2_, C, and NaCl was constructed with supercell parameters of a = b = 59.609 Å, c = 253.39 Å, and α = β = γ = 90°. The resulting structure was then imported into LAMMPS, with 4586 Cl atoms, 769 C atoms, 349 Ti atoms, 648 O atoms, and 4499 Na atoms for a total of 10,851 atoms, the composition of substances in the system was based on the raw material ratios in the chlorination process of the factory. Another system of pure NaCl was built at the same time, and the parameters of the pure NaCl system were a = b = 47.69 Å, c = 79.96 Å, α = β = γ = 90°, totaling 4896 atoms. Based on the actual experimental measurements of the pure NaCl density [17], the density of this system was set to be 1.30652 g/cm^3^, taking into account the influence of different temperatures and using the same potential function as in the reaction system, the simulation time was 6 ns with a time step of 1 fs. In LAMMPS, the parameters used were as follows: periodic boundary conditions with units set to metal; the conjugate gradient method for energy minimization with an energy tolerance of 1 × 10^−5^ eV/Å and force tolerance of 1 × 10^−5^ eV/atom; a maximum iteration step of 5000; and loading deep potential energy functions for structural relaxation and dynamic simulation calculations in LAMMPS. For DeePMD simulations at different temperatures (1073 K, 1173 K, 1273 K), the total time steps were set to be 4 ns with a time step length of 1 fs. The open-source software Open Visualization Tool (OVITO) 3.10.4 [40] was used for analyzing the results obtained from DeePMD simulations.

### 2.4. Experimental Details

**Figure 2:**
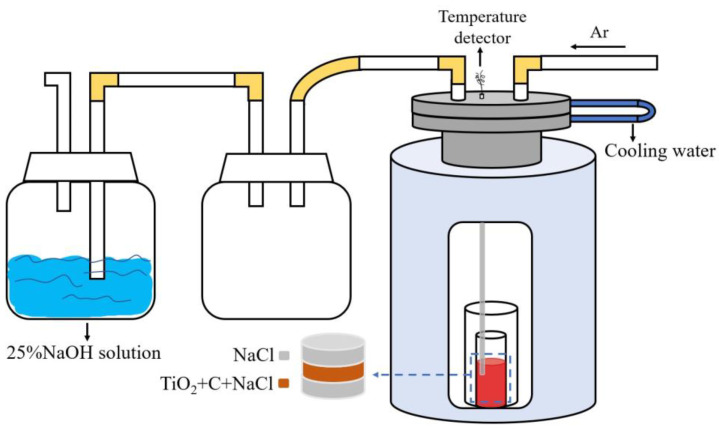
Refinement of experimental system design.

The equipment used for experimental verification is illustrated in Figure 2.

The raw materials employed in this experiment include TiO_2_ powder (purity greater than 99.8%), NaCl particles (purity greater than 99.8%), and carbon black (purity greater than 99.5%). Initially, the raw materials were mixed according to the proportions of 0.5 mol titanium dioxide, 1 mol sodium chloride, and 0.5 mol carbon black. Additionally, an excess amount of sodium chloride was placed at both the top and bottom of the mixing area. Subsequently, the resulting mixture was placed into a crucible and heated in a resistance furnace. The temperature inside the furnace increases at a rate of 10 °C per min. To ensure that the reaction had sufficient time to proceed, the internal temperature was maintained above 800 °C for a duration of one hour. During this period, the maximum temperature recorded by the temperature sensor reached 850 °C. Due to the potential temperature difference between the location of the reactants within the electric resistance furnace and that of the detector, the measured temperature is slightly elevated relative to the simulated temperature. Prior to heating, the argon gas was introduced into the system, and during the reaction process, the internal pressure of the system remained at standard atmospheric pressure.; argon (purity greater than 99.99%) was supplied at atmospheric pressure with a flow rate of 0.5 L/min. At the conclusion of the experiment, samples were removed after naturally cooling to room temperature. The composition of the post-reaction products underwent analysis via X-ray diffraction (XRD, Rigaku, Tokyo, Japan) as well as X-ray photoelectron spectroscopy (XPS, Thermo Fisher Scientific, East Grinstead, UK). In addition, inductively coupled plasma (ICP, PerkinElmer, Waltham, MA, USA) analysis was used to determine the titanium content of 25% NaOH solution after the end of the experiment, and to determine whether gaseous titanium chloride had formed in the chloride and was absorbed by sodium hydroxide solution during the experiment.

## 3. Results

### 3.1. AIMD

**Figure 3:**
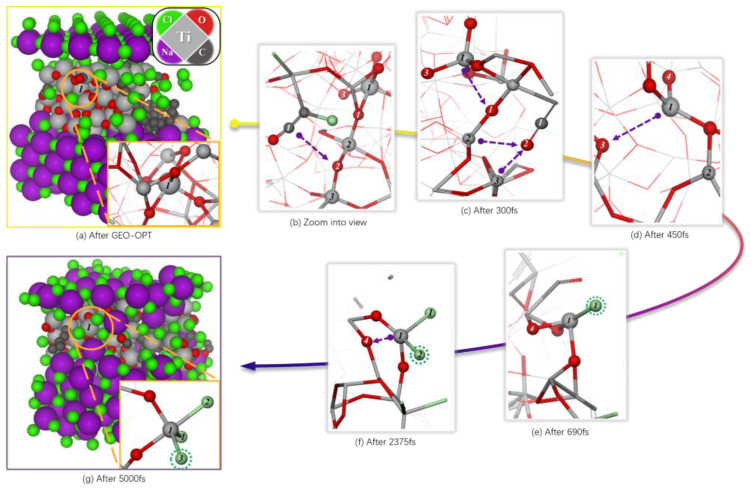
The TiO_2_-C-NaCl-Cl_2_ structure after geometric optimization and 5 ps dynamics simulation at 1073 K.

The optimized reaction system is shown in Figure 3a, the zoomed image of area 1 in Figure 3a is shown in Figure 3b. At 1073 K, within 0–300 fs, Figure 3b,c indicate that atom C1 gradually approached atom O2 which bridged atom Ti2 and atom Ti3 on the surface of the titanium dioxide, which led to the bonding between C1 and O2 and the detaching of O2 from its original bridging position between Ti2 and Ti3, forming an oxygen vacancy defect, and the bond lengths of Ti2-O2 and Ti3-O2 changed from the optimized 1.84217 Å and 1.83314 Å to 3.04041 Å and 2.87562 Å, respectively. Table 1 shows that there was no population overlap between these two atoms. Additionally, the bond length between C1 and O2 changed from 3.68690 Å to 1.35759 Å, with a bond population of 0.80 at 300 fs (Table 1).

**Figure 4:**
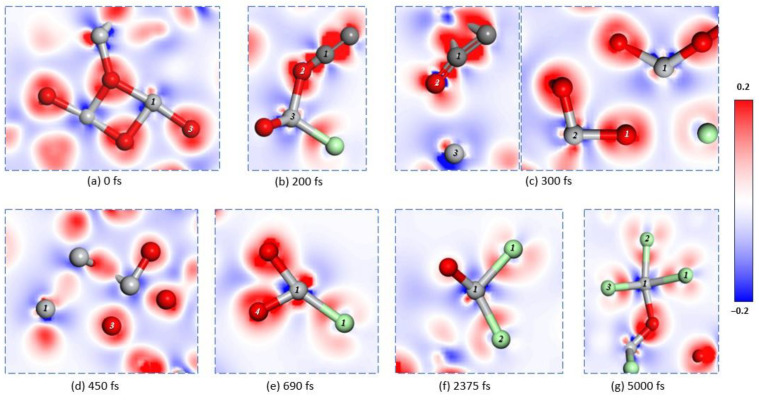
Localized electron difference density in the system at different times.

Furthermore, the electron density difference in Figure 4b,c shows a strong covalent bond formed between C1 and O2, while the original Ti3-O2 ionic bonds have been broken. The disconnection of O2 from Ti2 strengthened the bonding between Ti2 and nearby O1, with the population increasing from 0.40 to 0.48. Additionally, the bond length of Ti1-O1 changed from 2.03791 Å to 2.95439 Å as its population shifted from 0.31 to 0.02 (Table 1). The electron density difference indicates that Ti1 and O1 are no longer bonded (Figure 4c).

At a simulation time of 450 fs, the bond length of Ti1-O3 increased from 1.98639 Å to 3.4442 Å, with no bond population present (Table 1). The electron density difference between Ti1 and O3 (Figure 4d) indicates that the original bonding has been broken, similar to O2 (Figure 3d). Following the breakage of bonds between Ti1 and O1 as well as O3, Ti1 gradually moves from within the optimized titanium dioxide crystal (Figure 3a) to its surface (Figure 3d). At this point, in an unsaturated state, Cl1 approaches Ti1 when the simulation time reaches 690 fs and successfully adsorbs onto it (Figure 3e). The bond length and population for Ti1-Cl1 are measured at 2.34211 Å and 0.54, respectively (Table 1), indicating an ionic covalent bond formation. Furthermore, the electron density difference for Ti1-Cl2 shows a significant electron transfer trend from Ti to Cl compared with O (Figure 4e). By the time that the simulated time reaches 2375 fs, the ionic covalent bond between Ti1 and O4 breaks (Figure 3f), with its bond length increasing from 1.87781 Å to 2.49343 Å while the population decreases from 0.45 to 0.11 (Table 1). Due to this separation based on electron density differences shown in Figure 3f along with a population of 0.40 and a bond length of 2.32773 Å for Ti-Cl2, we can infer that another ionic covalent bond exists between them (Table 1). By reaching a simulation time of 5000 fs, ionic covalent bonds form between Ti-Cl pairs: Cl1, Cl2, and Cl3 are observed in Figure 3g. For instance, the parameters for Ti1-Cl3 show a population of 0.65 and a bond length of 2.21200 Å, respectively (Table 1). At this stage (5000 fs), Ti is not yet fully chlorinated (Figure 4g).

In Figure 4, the atomic-level transformation of titanium dioxide into titanium tetrachloride in molten salt with carbon chloride is shown. Reaction kinetics indicate that fluidized bed chlorination based on C and Cl principles leads to specific conclusions. The analysis of electronic energy properties shows that Cl2 adsorption on TiO_2_ is physical. It is known that during chlorination, carbon reduces the oxygen potential of titanium dioxide crystals, corresponding to Cl1 and Cl2 being provided by adjacent Ti atoms. As C removes O from TiO_2_, the process shifts from physical to chemical adsorption, forming a strong ionic covalent bond between Ti and Cl. The formation of CO as C extracts O destabilizes the structure. Reaction kinetics suggest that C lowers oxygen potential [11], resulting in unsaturated low-valence Ti and various degrees of chlorinated titanium oxide (TiO_x_Cl_4−x_). In the research conducted by Julio et al. [11], it was observed that during fluidized bed chlorination experiments, the weight of the reactant initially increased and then decreased. Based on molecular dynamics analysis, we can explain this weight change: Chlorine gas readily adsorbs onto TiO_2_ after structural optimization, but the reaction rate between C and TiO_2_ is relatively slow. After some time, a significant amount of the gaseous product is no longer included in the measured weight, leading to a rapid decrease. This occurs because many Ti atoms adsorbed on the surface have been consumed, preventing further binding with chlorine gas and resulting in reduced system weight.

The behavior of chlorine gas in boiling chlorination reactions is well established. Previous studies have addressed intermediate chlorination products in the fluidized process, but there are few reports on the form and transformation mechanism of chlorine gas in molten salt chlorination. Therefore, this study investigates the behavior of chlorine gas in molten salt reaction systems.

**Figure 5:**
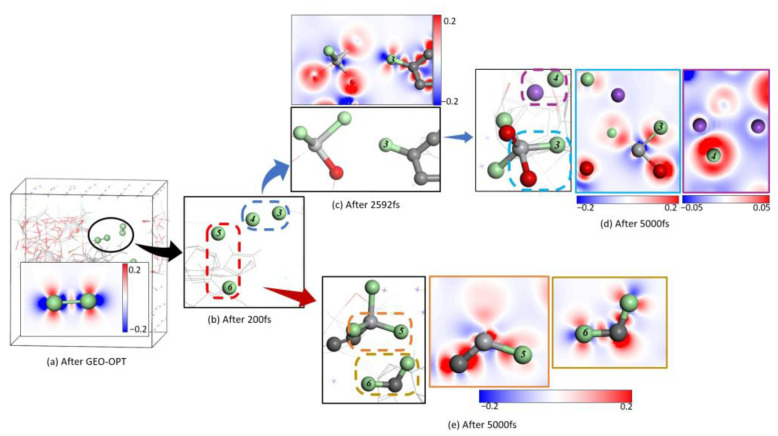
The presence form and conversion pathways of chlorine in the system.

After optimization, there are still multiple chlorine molecules in the system. According to the electron density difference in Figure 5a, it is evident that there exist covalent bonds between the chlorine atoms.

It is evident from the trajectories of the two chlorine gas molecules depicted in Figure 5 that both Cl3 and Cl5 have formed bonds with Ti atoms. However, during the course of this study, it was observed that the behavior of Cl4 warrants greater attention. The Cl4 atom integrated into NaCl during the dissociation process of chlorine gas molecules. From an analysis of the electron distribution, it was clear that an ionic bond had been established; thus, chlorine atoms from the gaseous state had contributed to NaCl formation. This finding also provides new insights for future research directions.

Considering the high computational cost associated with AIMD, the limitations on the number of atoms in the system, and the complexity of reaction systems, subsequent research will continue to explore these aspects through deep learning using DeePMD.

### 3.2. DeePMD

**Figure 6:**
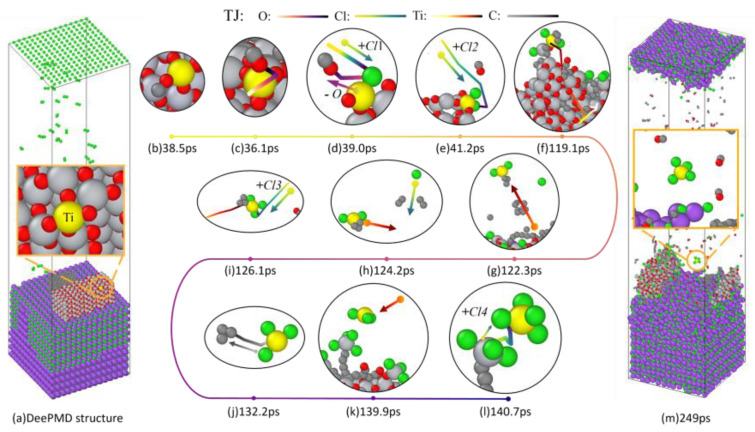
DeePMD structure over time at 1073 K.

The open-source software LAMMPS was used to conduct DeePMD simulations based on the trained deep potential. The results of DeePMD simulation are shown in Figure 6, where the Ti atoms are marked in yellow for emphasis. In order to make the atomic movements clearer, the trajectories of each atom within a certain period of time were highlighted using OVITO software.

Figure 6a reveals the structural model imported into LAMMPS for DeePMD calculations.

At 38.5 ps, it can be observed that a C atom is in contact with a bridging oxygen atom connected to Ti (Figure 6b). At 36.1 ps, it is evident that the chemical adsorption of C causes the bridging O to no longer reside between the two Ti atoms (Figure 6c). By observing the trajectory at 39 ps, it is apparent that due to the absence of O, the first Cl atom adsorbs onto Ti (Figure 6d). The process of CO generation is abundant in the system as depicted in Figure 6b–d, with a significant presence of CO observed in Figure 6m. As confirmed in the AIMD results section, when the titanium dioxide crystal loses the bridging O due to C interacting within its internal structure, the crystal gradually discards peripheral Ti in order to attract external O towards its interior to maintain stability, thus leaving peripheral Ti atoms unsaturated and facilitating the adsorption of free Cl atoms onto them, as seen at a simulation time of 41.2 ps where a second chlorine atom is attached to the labeled Ti (Figure 6e). As the reaction progresses, the crystal gradually disintegrates. By the simulated time of 119.1 ps, the labeled Ti atoms are no longer connected to the main body of the titanium dioxide crystal (Figure 6f). Furthermore, at a simulation time of 122.3 ps, the labeled Ti atoms form a small molecule with two Cl atoms and a short carbon ring, entering into a free state (Figure 6g). As shown in Figure 6h, the labeled Ti atom is about to adsorb a free Cl atom in the direction indicated by arrow, and by a simulation time of 126.1 ps, there are already three Cl atoms connected to it. After three Cl atoms have attached to Ti, there is an obvious weakening in interaction between Ti and C, leading to the detachment of a short carbon ring from Ti by simulation time 132.2 ps which became a titanium trichloride molecule (Figure 6j). At a simulated time of 139.9 ps, the free titanium trichloride approached a tetrachloride titanium molecule captured by a long carbon chain (Figure 6k). Since there were no other atoms pulling its charge near the labeled Ti atom, some of the electrons of the originally formed tetrachloride titanium were attracted away by the long carbon chain, resulting in a weaker force on the Cl atoms compared with the labeled Ti atom. At this point, four Cl atoms are connected around the labeled Ti atom, indicating the formation of tetrachloride titanium (Figure 6m) as the subject of this study.

The simulation system, scaled up using the DeePMD method, is particularly suitable for quantitative analysis of the chlorination degree, bond lengths, quantities, and their temporal relationships in TiO_2_ through the radial distribution function [41] (RDF). The RDF serves as a tool to describe the spatial distribution of particles within a system. Its principle involves selecting a specified atom as the center and examining the distribution of other atoms at a distance r from this central atom. Mathematically, it represents the ratio of local number density at position r to the overall number density. In this context, it can provide insights into the relationship between Ti-Cl bonds to some extent.

**Figure 7:**
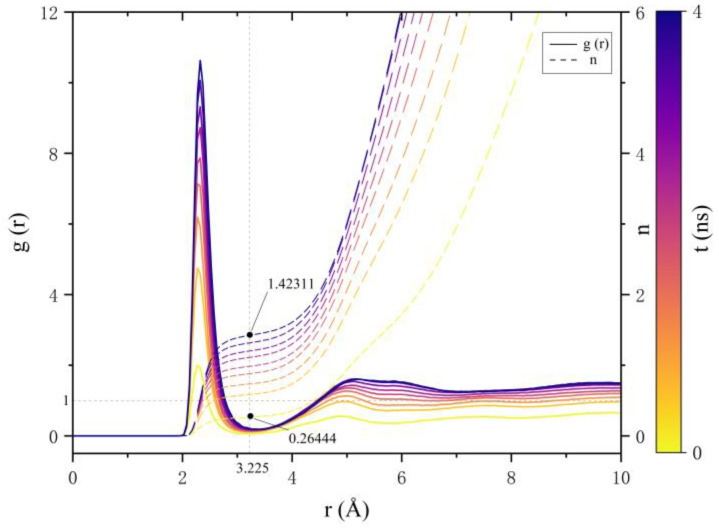
Radial distribution function of Ti-Cl in the 1073 K system.

By comparing variations in the RDF over different time periods (Figure 7), it was observed that the bonding probability between Ti and Cl gradually increases with time, resulting in a significant rise in the coordination number of Ti-Cl. Table 2 presents the coordination numbers between Ti and Cl at various stages. This phenomenon indicates a marked increase in the chlorination degree of TiO_2_ within the simulated system.

**Figure 8:**
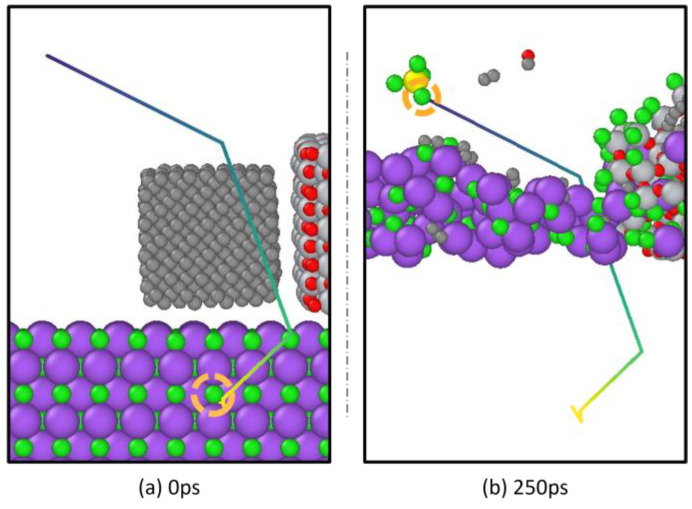
Comparison of the 0 ps and 250 ps positions of chlorine atoms on titanium tetrachloride.

Figure 8 exhibits the atomic motion trajectory of the chlorine atom connecting to the fourth labeled Ti atom from 0 ps to 249 ps.

By comparing the positions of the same chlorine atom in the system at 0 ps and 249 ps in Figure 8a,b, it is apparent that the fourth chlorine atom in the chlorination process originates from the molten salt. According to the previously described mechanism of molten salt chlorination reduction and in combination with Figure 6l, it can be inferred that this chlorine atom first forms a Ti-Cl ionic covalent bond with an unmarked Ti atom, as shown in Figure 6l. Subsequently, under the specific environmental influences mentioned above, it ultimately becomes connected to a labeled Ti atom as part of its coordination sphere.

According to the simulation results obtained from DeePMD, it was observed that NaCl can participate in the chlorination process of TiO_2_. In light of this finding, this study will design validation experiments to ensure that the simulated results align with actual occurrences.

### 3.3. Experimentation and Validation

**Figure 9:**
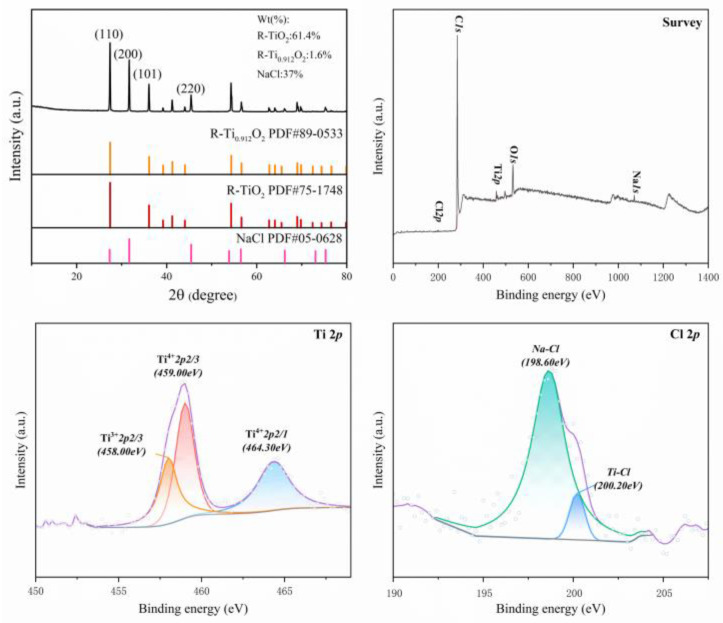
XRD and XPS detection results of the products.

In light of the computational results mentioned earlier, we proceeded with verification experiments using the experimental system previously described. By analyzing the XRD and XPS detection results of the products (Figure 9), we found a small amount of unsaturated low-valent titanium oxide compounds present in the samples.

Furthermore, an analysis of the 2p orbitals for Ti and Cl elements indicated a noticeable degree of mild chlorination within the products. Additionally, by extracting a small sample from a 25% NaCl solution for ICP detection on Ti elements in the experiment, a concentration of 0.03 mg/L of titanium was detected. Given that titanium oxides have extremely high boiling points, it is unlikely that they could vaporize within the temperature range designed for this study. Therefore, the products transported by argon and mixed with the NaOH solution are expected to be gaseous titanium chlorides. This indicates that NaCl facilitates the reaction of titanium oxides through the aforementioned mechanism. This finding indirectly confirms the reliability of simulation results observed via the DeePMD method, which suggest that NaCl can participate in chlorination reactions during the chlorination process. These experimental findings align well with our computational simulations, providing compelling evidence that NaCl can serve as a source of chlorine during chlorination reactions when utilizing molecular dynamics methods. This demonstrates that chlorine atoms for producing titanium tetrachloride do not necessarily come from chloride gas; they can also be obtained from molten salt under special conditions.

**Figure 10:**
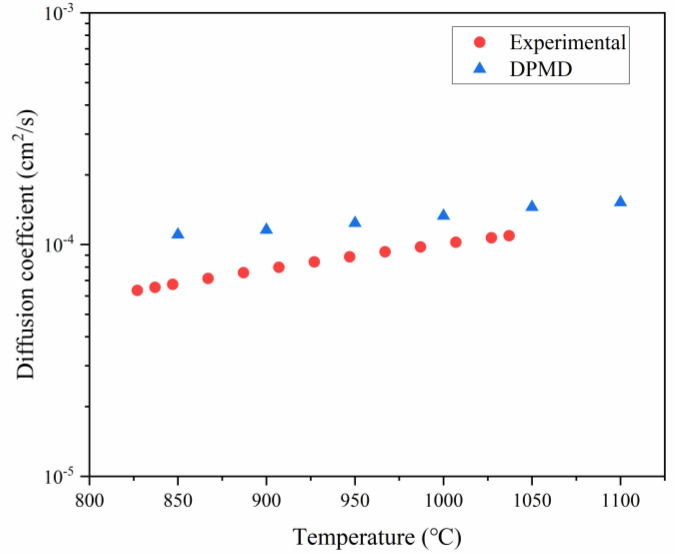
Comparison of chloride ions self-diffusion coefficients predicted by DeePMD with experimental data.

The theoretical values predicted by the DeePMD method have inherent limitations, as their accuracy is closely related to the precision of the dataset used for training and various weights in the training process. Additionally, data obtained through experimental methods may be influenced by factors such as instrument precision and the purity and ratio of raw materials. Therefore, it is essential to select appropriate research methods tailored to different research objectives. The simulated data in Figure 10 were analyzed using interpolation methods to calculate the standard deviation values at a constant temperature, compared with the experimental data. The overall standard deviation is found to be 9.58 × 10^−6^ (for more details about SD see the Supplementary File).

As mentioned before, the chlorination reaction of molten salt as a complex exothermic heterogeneous (gas–liquid–solid) interfacial reaction, combined with the conversion pathway in the chlorine system pointed out in this paper, reacts with solids on the one hand, and, on the other hand, it can replenish chloride ions in the molten salt. Since the self-diffusion coefficient of chloride ions in molten salt is also closely related to the reaction efficiency, this paper also investigates the self-diffusion coefficient of chloride ions in sodium chloride by calculating the self-diffusion coefficient of chloride ions according to the linear fitting by the calculation of the mean square displacement of chloride ions, and compares with the data measured by experimental means by previous researchers. Therefore, in this study, the theoretically simulated chloride diffusion coefficients at intervals of fifty degrees from 850 to 1100 °C are calculated using the constructed NaCl model and compared with the experimental data. In Figure 10, the blue dots represent theoretical values of Cl^−^ ion diffusion coefficients at different temperatures calculated using DeePMD method, while the red dots represent fitted values of Cl^−^ ion self-diffusion coefficients in molten NaCl within the temperature range from 827 °C to 1037 °C that were experimentally measured [18]. The comparative results indicate that, taking into account factors such as errors and methodological limitations, the theoretical values obtained through the DPMD method can predict a closely related result.

## 4. Conclusions and Discussion

In the AIMD section, introducing carbon elements initially removed the bridging oxygen that stabilizes titanium dioxide, leading to gradual structural destabilization. This supports traditional kinetic views, indicating that carbon addition can lower oxygen potential. Thermodynamic calculations also suggest that low-valent titanium oxides are more reactive than high-valent ones. However, while gradual chlorination of Ti has been observed, limitations in the AIMD method hinder a comprehensive understanding of the chlorination mechanism. Additionally, we tracked chlorine gas dissociation within the reaction system, and analysis of its electronic energy properties revealed that chlorine gas can dissociate into chloride ions, supplemented into NaCl. In light of this discovery, future investigations into the viscosity of NaCl and the solubility of chlorine gas within NaCl will unveil further details regarding its reaction kinetics. This consideration may also extend to the study of molten salts such as KCl and CaCl_2_.

In the DeePMD section, a complete transformation process from titanium dioxide to titanium tetrachloride was observed. By tracking the trajectories of chlorine atoms on fully generated titanium tetrachloride, it is evident that sodium chloride can provide chlorine atoms during the chlorination process. Based on this conclusion, relevant experiments were designed for validation, and the experimental results aligned well with the simulation outcomes. Furthermore, by comparing diffusion coefficients of chloride ions using statistical mean square displacement methods simulated with DeePMD at various temperatures, it was found that these results are consistent with previously measured experimental data regarding chloride ion diffusion coefficients in pure NaCl reported by other researchers. This indicates that the DeePMD method can serve as a novel characterization technique when conventional means fail to yield results in complex reaction systems.

In conclusion, the DeePMD method based on deep learning algorithms can significantly expand the scale and time scale of AIMD simulations in this paper, while maintaining DFT-level accuracy. This expansion allows for a more complete understanding of the complex reactions that are difficult to fully reveal in AIMD simulations. Through the analysis and summarization of atomic interactions in AIMD using deep learning algorithms, some phenomena that are often overlooked in AIMD simulations have been revealed. The DeePMD method provides an unprecedented level of detail for atomic-level understanding of each reaction process, extracting detailed reaction mechanisms from atomic motion trajectories. This goes beyond what can be achieved in laboratory experiments and enables researchers to better understand reaction mechanisms. To enhance the chlorination efficiency of reactions, it is essential to understand the underlying reaction mechanisms. This study employs the DeePMD method to comprehensively elucidate the mechanism by which titanium dioxide is converted into titanium tetrachloride. Further research is necessary to appropriately utilize these reaction mechanisms for improving chlorination efficiency. An increase in chlorination efficiency implies that a reduced amount of waste salt can be generated while producing the same quantity of product, thereby mitigating environmental pollution.

## Figures and Tables

**Table 1:** Bond lengths and population of the system at 1073 K.

Bond	After OPT		300 fs		450 fs		690 fs		2375 fs		5000 fs	
	Population	Length (Å)	Population	Length (Å)	Population	Length (Å)	Population	Length (Å)	Population	Length (Å)	Population	Length (Å)
C1-O2	—	3.68690	0.80	1.35759	0.59	1.38324	0.74	1.27884	—	2.86763	—	—
Ti2-O1	0.41	1.88325	0.48	1.97810	0.48	2.03756	0.39	1.91971	0.40	2.01386	0.43	1.92439
Ti2-O2	0.45	1.84217	—	3.04041	—	—	—	—	—	—	—	—
Ti3-O2	0.50	1.83314	—	2.87562	—	—	—	—	—	—	—	—
Ti1-O1	0.31	2.03791	0.02	2.95439	—	—	—	—	—	—	—	—
Ti1-O3	0.30	2.11359	0.36	1.98639	—	3.44442	—	—	—	—	—	—
Ti1-O4	0.20	2.17955	0.54	1.85613	0.43	1.87039	0.45	1.87781	0.11	2.49343	—	4.19313
Ti1-Cl1	—	—	—	—	—	—	0.54	2.34211	0.55	2.37071	0.51	2.29606
Ti1-Cl2	—	—	—	—	—	—	—	—	0.40	2.32773	0.43	2.26223
Ti1-Cl3	—	—	—	—	—	—	—	—	—	—	0.65	2.21200

**Table 2:** The coordination number (CN) between Ti-Cl bonds in the 1073 K system during each time period.

Time Period (ns)	CN	Time Period (ns)	CN
0~0.2	0.26444	1.2~1.5	1.11075
0.2~0.5	0.58524	1.5~2	1.20147
0.5~0.7	0.73463	2.5~3	1.31607
0.7~1	0.87946	3.9~4	1.42311
1~1.2	0.98397		

## Data Availability

The data that support the findings of this study are available on request from the corresponding author, [X.C.], upon reasonable request.

## Supplementary Materials

The following supporting information can be downloaded at: https://www.mdpi.com/article/10.3390/ma18030659/s1, Table S1: The absolute error calculated from simulated data, referenced against experimental data.
