# Supplementary material for: Modeling the Carbothermal Chlorination Mechanism of Titanium Dioxide in Molten Salt Using a Deep Neural Network Potential
Source: Materials (Basel). 2025 Feb 2;18(3):659. doi: 10.3390/ma18030659 (PMC11820525; doi:10.3390/ma18030659)
Supplement: Supplementary file 1 [file materials-18-00659-s001.zip › materials-3434939-supplementary.pdf]

Standard deviation:

$$y_{\text{sim}} = y_1 + \frac{(T_{\text{exp}} - T_1)(y_2 - y_1)}{T_2 - T_1}$$

$$\text{Absolute error} = y_{\text{sim}} - y_{\text{exp}}$$

$$\bar{\epsilon} = \frac{1}{N}\sum_{i=1}^{N} \epsilon_i$$

$$\text{Variance} = \frac{1}{N-1}\sum_{i=1}^{N} (\epsilon_i - \bar{\epsilon})^2$$

$$\text{Standard deviation} = \sqrt{\text{Variance}}$$

- T_{exp}: Experimental temperature point.
- T_1, T_2: Nearest simulated temperature points bracketing Texp.
- y_1, y_2: Simulated values at T_1 and T_2.
- y_{sim}: Interpolated simulated value at T_{exp}.
- y_{exp}: Experimental value.
- y_{sim}: Interpolated simulated value
- ϵ_i: Absolute error for the i-th data point.
- N: Number of data points.

S-table.1 The absolute error calculated from simulated data, referenced against experimental data.

Temperature	Self-diffusion coefficients (experimental value cm^2/s)	Self-diffusion coefficients (interpolated simulated value cm^2/s)	Absolute error
1100K	6.35×10^{-5}	9.62×10^{-5}	3.27×10^{-5}
1110K	6.54×10^{-5}	1.02×10^{-4}	3.68×10^{-5}
1120K	6.74×10^{-5}	1.08×10^{-4}	4.08×10^{-5}
1140K	7.15×10^{-5}	1.20×10^{-4}	4.87×10^{-5}
1160K	7.57×10^{-5}	1.32×10^{-4}	5.65×10^{-5}
1180K	7.99×10^{-5}	1.22×10^{-4}	4.17×10^{-5}
1200K	8.42×10^{-5}	1.38×10^{-4}	5.34×10^{-5}
1220K	8.86×10^{-5}	1.54×10^{-4}	6.50×10^{-5}
1260K	9.32×10^{-5}	1.39×10^{-4}	4.61×10^{-5}
1280K	9.77×10^{-5}	1.57×10^{-4}	5.96×10^{-5}
1300K	1.02×10^{-4}	1.41×10^{-4}	3.94×10^{-5}
1300K	1.07×10^{-4}	1.65×10^{-4}	5.84×10^{-5}
1310K	1.09×10^{-4}	1.77×10^{-4}	6.84×10^{-5}

The standard deviation of the absolute error between the simulated values and the experimental values is 9.58×10^{-6}
